# Study of Fungal Colonization of Wheat Kernels in Syria with a Focus on *Fusarium* Species

**DOI:** 10.3390/ijms14035938

**Published:** 2013-03-14

**Authors:** Dima Alkadri, Paola Nipoti, Katharina Döll, Petr Karlovsky, Antonio Prodi, Annamaria Pisi

**Affiliations:** 1 Department of Agroenvironmental Science and Technology, Alma Mater Studiorum Bologna University, viale G. Fanin 44, Bologna 40127, Italy; E-Mails: dima.alkadri2@unibo.it (D.A.); paola.nipoti@unibo.it (P.N.); annamaria.pisi@unibo.it (A.P.); 2 Molecular Phytopathology and Mycotoxin Research, University of Göttingen, Grisebachstrasse 6, Göttingen 37077, Germany; E-Mails: kdoell@gwdg.de (K.D.); pkarlov@gwdg.de (P.K.)

**Keywords:** Syria, wheat kernels, *Fusarium* chemotypes, trichothecenes

## Abstract

Wheat is one of the main crops in Mediterranean countries, and its cultivation has an important role in the Syrian economy. In Syria, *Fusarium* head blight (FHB) has not been reported so far. Mycological analysis of 48 samples of wheat kernels collected from cultivation areas with different climatic conditions were performed in 2009 and 2010. Fungal isolates were identified at the genus level morphologically; *Fusarium* species were characterized morphologically and by species-specific PCR. The most frequent fungal genera found were *Alternaria* spp. and *Cladosporium* spp., with frequencies of 24.7% and 8.1%, respectively, while the frequency of *Fusarium* spp. was 1.5% of kernels. Most frequent *Fusarium* species were *F. tricinctum* (30% of all *Fusarium* isolates), *F. culmorum* (18%), *F. equiseti* (14%) and *F. graminearum* (13%). The mycotoxin production potential of selected *Fusarium* isolates was assessed by HPLC-MS analysis of rice cultures; chemotyping by PCR was carried out for comparison. All six *F. graminearum* strains tested produced small amounts (<3 mg/kg) of nivalenol (NIV). All ten *F. culmorum* strains tested produced large amounts of trichothecenes (>100 mg/kg); four strains produced NIV and six strains produced deoxynivalenol (DON) and 3-acetyl-deoxynivalenol (3Ac-DON). PCR chemotyping lead to an oversimplified picture, because all 3Ac-DON chemotype strains produced more DON than 3Ac-DON; furthermore, the strongest NIV producers produced significant amounts of DON. All tested strains of *F. culmorum*, *F. graminearum*, *F. pseudograminearum* (two strains) and most *F. equiseti* strains (five of six strains) produced zearalenone. Grains of durum wheat were more frequently colonized by *Fusarium* spp. than grains of soft wheat. Incidence of *Fusarium* spp. in irrigated fields was higher than in rainfed fields. The incidence of *Fusarium* strains producing mycotoxins raises concerns about the risk of *Fusarium* head blight to Syria and its consequences for public health.

## 1. Introduction

**Figure 1:**
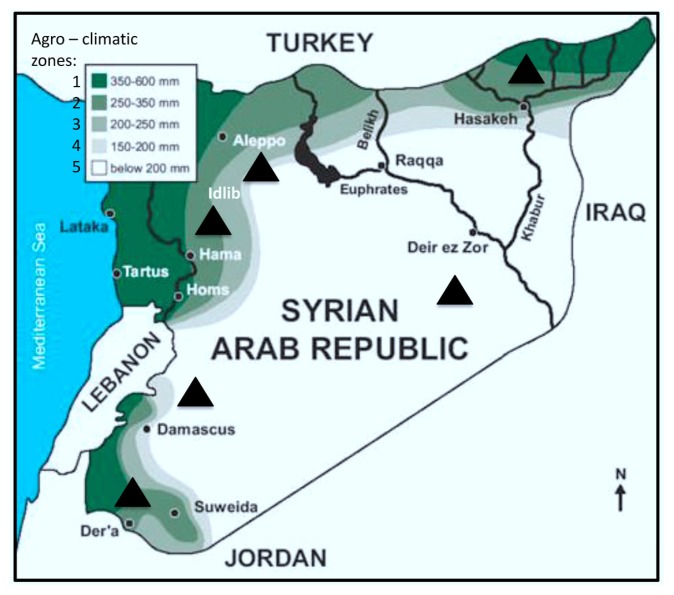
Agro-climatic zones in Syria based on rainfall (in mm) [3], and provinces where wheat samples were collected (▲).

Durum wheat constitutes the largest part of the staple food in the southern Mediterranean countries [1]. In Syria, wheat cultivation covers 83% of the cultivated area and has a central role in the diet [2]. There are five distinct agro-climatic zones in Syria based on rainfall (Figure 1) [3]. Wheat is present in all these areas. Rainfed wheat is concentrated in high rainfall zones 1 and 2, while irrigation is necessary in zones 3, 4 and 5. The largest zones are 5, 1 and 2, covering 53%, 42% and 40% of the total cultivated land, respectively [4].

Syria produces both durum and soft wheat over the winter season. Soft wheat is cultivated mainly in irrigated areas, durum wheat in rainfed areas. Depending on the rainfall, rainfed wheat yields are highly unstable, with the average ranging from less than 0.5 tons per ha in a drought year to over 1.7 tons per ha in a year of good rainfall. Yields are more stable in irrigated areas, with the national average ranging from 3.0 to 4.4 tons per ha [2]. Syrian agricultural policy resulted in an increase in cultivation of wheat in the irrigated land from 229,000 ha in 1988 to 800,000 ha in 2003 and 1.9 million ha in 2005 [4].

The seed infection by *Fusarium* pathogens is a great risk for wheat cultivation. *Fusarium* species are a widespread pathogenic fungi, which can cause *Fusarium* head blight (FHB) and *Fusarium* crown rot in wheat [5]. *Fusarium* crown rot is known to occur in Syria; *Fusarium* isolates recovered from the subcrown internode of durum wheat were identified as *F. avenaceum*, *F. culmorum*, *F. equiseti*, *F. graminearum* and *F. poae*[6]. FHB or scab, which is a more dangerous and important disease, has not been reported in Syria yet.

The etiology of FHB is complex, due to the involvement of several species of *Fusarium* and *Microdochium nivale* (Fr.) Samuels & I.C. Hallett. The *Fusarium* species most frequently isolated from wheat ears worldwide are *Fusarium graminearum* Schwabe (teleomorph *Gibberella zeae* (Schwein.) Petch), *F. culmorum* (W.G. Sm.) Sacc., *F. avenaceum* (Fr.) Sacc. (teleomorph *Gibberella avenacea* R.J. Cook) and *F. poae* (Peck) Wollenw [7].

The distribution of *Fusarium* species in wheat and their dominance over other fungi is affected by climatic conditions (temperature, humidity, *etc*.), agricultural practices (soil tillage, crop rotation, nitrogen fertilizers, pesticide treatment, *etc*.) and cultivar susceptibility [4]. FHB causes high yield losses [4]; in addition, colonization of wheat with *Fusarium* species can cause the contamination of grain with toxic fungal secondary metabolites (mycotoxins) that are recognized as health hazards for both human and farm animals [8].

Toxigenic *Fusarium* species produce several mycotoxins, such as trichothecenes A and B, zearalenone, moniliformin, depsipeptides and fusaric acid. Most attention in the analysis of FHB-afflicted wheat grains has so far been devoted to deoxynivalenol (DON), acetylated forms of DON (3Ac-DON and 15Ac-DON), nivalenol (NIV), fusarenon X (Fus X) and zearalenone (ZEN) [7]. The knowledge of the occurrence of *Fusarium* species in different growing areas helps to predict the mycotoxin content of harvested grain. The maximum allowed limits for mycotoxin levels in food have been established for the protection of the consumer. For instance, limits for DON and ZEN in food exist in Europe (EU-regulation 1881/2006).

In addition to *Fusarium* spp. in FHB, other fungi, e.g., *Alternaria* spp., *Cladosporium* spp., *Epicoccum* spp. and *Rhizopus* spp., infect wheat grain in the field, causing quality loss, due to undesirable color and odor. In Syria, little information is available on fungi associated with durum and soft wheat grain and their distribution in the main production area.

The aims of the present research were (a) to study the diversity of fungal species colonizing wheat kernels from different provinces of Syria; (b) to determine the incidence of mycotoxin-producing *Fusarium* species; (c) to estimate the potential of selected *Fusarium* isolates to produce mycotoxins; (d) to compare infection frequencies of durum and soft wheat by *Fusarium* spp. and (e) to compare irrigated and rainfed fields regarding *Fusarium* spp. incidence.

## 2. Results and Discussion

**Figure 2:**
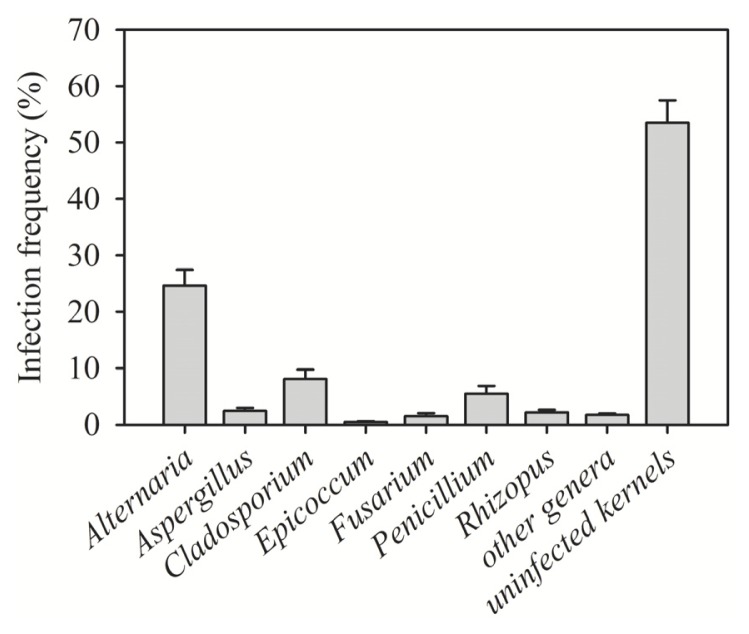
Frequency of the major genera of fungi contaminating wheat kernels in Syria in 2009–2010. * Other genera = *Absidia*, *Chaetomium*, *Cylindrocarpon*, *Helminthosporium*, *Nigrospora*, *Phoma*, *Sclerotinia*, *Septoria* and *Stemphylium*. * Error bars represent the standard error of mean.

In the surveys carried out in 2009 and 2010 on durum and soft wheat grains collected from different provinces in Syria, 17 different genera of fungi were identified in surface-disinfected kernels. *Alternaria* spp. and *Cladosporium* spp. were the most frequent, with an isolation frequency of 24.7% and 8.1%, respectively (Figure 2). High kernel infection with *Alternaria* and *Cladosporium* was detected in the samples collected from all six provinces investigated (Table 1). These saprophytic fungi are known to cause grey or black discoloration of heads and seeds, resulting in sooty molds, black points or smudge, and under certain conditions, they may produce mycotoxins. The prevalence of *Alternaria* and *Cladosporium* among fungal genera was in agreement with a study from Iran [9]. Storage fungal genera, *Penicillium* and *Aspergillus*, were isolated at frequencies of 5.5% and 2.4%, respectively (Figure 2).

**Figure 3:**
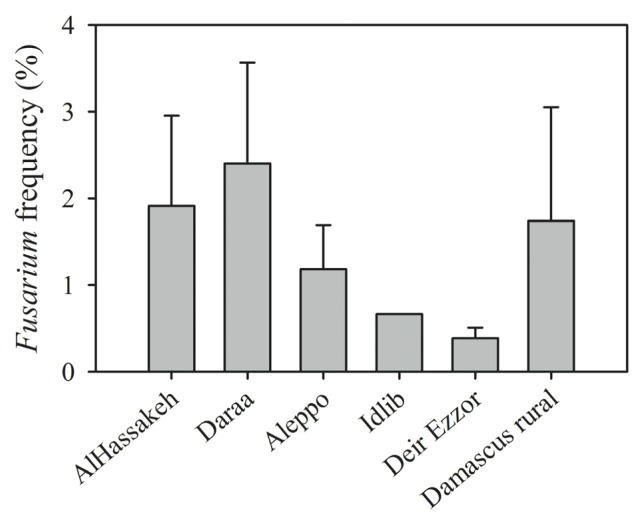
Incidence of *Fusarium* spp. in wheat kernels sampled from different provinces in Syria in 2009–2010. * Error bars represent the standard error of mean.

*Fusarium* spp. was present in 62.5% of all samples (Table 2) with a frequency of 2.4% (minimum 0.25%, maximum 25%). *Fusarium* species were mostly isolated from samples collected in the Daraa (agro-climatic zone 1, 2 and 3) and Al-Hassakeh (on the border between agro-climatic zones 1 and 2) areas, with frequencies of 2.4% and 1.9%, respectively, while the lowest relative frequency (0.4%) was in Deir Ezzor, a very dry region (agro-climatic zone 5; Figure 3). The differences in the incidence of *Fusarium* spp. among provinces can likely be accounted for by differences in their climate.

The incidence of *Fusarium* species causing FHB was likely underestimated, since severely infected and shriveled kernels, which are very light in weight, are expelled with the chaff during combined harvesting [10]. FHB has not been reported in Syria yet.

**Figure 4:**
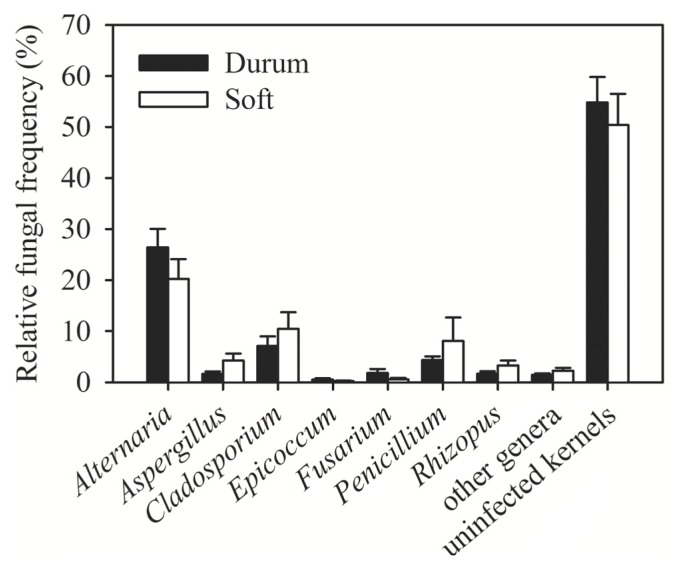
Incidence of fungal genera in durum and soft wheat kernels in Syria in 2009–2010. * Error bars represent the standard error of mean.

Promising sources of resistance to FHB have recently been identified in Syrian durum landraces [11,12]. Because durum wheat is more susceptible to FHB than soft wheat [13], we compared the incidence of *Fusarium* spp. in durum and soft wheat cultivars in Syria (Figure 4). Durum wheat was more often colonized by *Fusarium* spp. (frequency 2%) than soft wheat (frequency 1%). Colonization rates of durum and soft wheat cultivars by other fungal genera were comparable; the total colonization rates by all fungi were similar, too (Figure 4).

**Figure 5:**
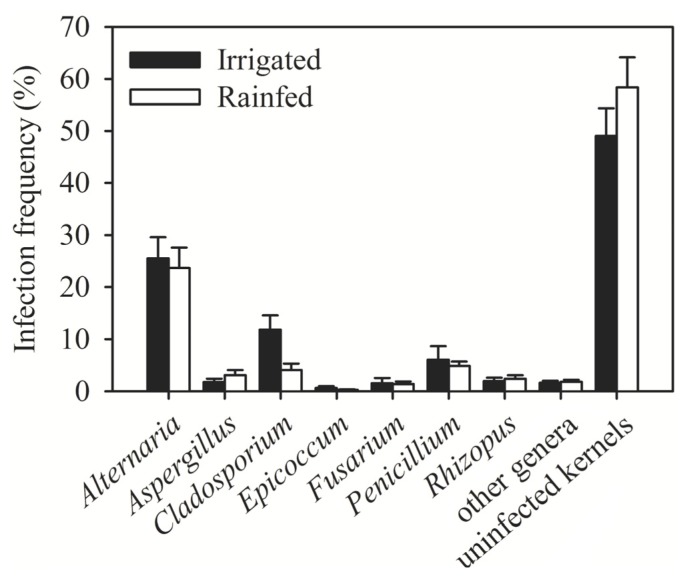
Incidence of fungal genera in wheat kernels collected from irrigated and rainfed fields. * Error bars represent the standard error of mean.

High humidity during flowering is one of the key factors promoting FHB [5]. The trend toward higher irrigation rates after the drought waves, which hit Syria recently, might therefore have increased the risk of FHB. We studied this question by comparing the incidence of fungi in kernel samples collected from irrigated and rainfed fields. Irrigated fields had a higher incidence of *Fusarium* spp. than rainfed fields (2% *vs.* 1%). Colonization of grains by other fungal genera appeared slightly higher in irrigated fields, too, and the same trend was reflected by the total colonization by all fungal genera (Figure 5).

A total of 163 *Fusarium* isolates were morphologically identified to the species level. Species-specific PCR was used to verify the assignments of selected isolates. Genomic DNA of strains putatively identified as *F. culmorum* (11 strains), *F. graminearum* (six strains), *F. equiseti* (nine strains), *F. proliferatum* (four strains) and *F. verticillioides* (five strains) and two isolates of *F. pseudograminearum* were used in this analysis. The products of DNA amplification with species-specific primers (see Experimental Section) were about 570 bp for *F. culmorum*, 400 bp for *F. equiseti*, 450 bp for *F. graminearum*, 585 bp for *F. proliferatum*, 578 bp for *F. verticillioides* and 523 bp *F. pseudograminearum*. These sizes correspond to published values for species-specific PCR products, confirming the morphological identification.

Morphological and molecular data revealed that the prevalent *Fusarium* species isolated were *F. tricinctum*, *F. culmorum*, *F. graminearum*, *F. equiseti*, *F. verticillioides* and *F. proliferatum*, with relative frequencies of 30.1%, 17.8%, 12.9%, 14.1%, 10.4% and 8.0%, respectively. *F. semitectum*, *F. pseudograminearum* and *F. oxysporum* were present in low frequencies of 1.8%, 1.8% and 3.0%, respectively.

Our study indicates the dominance of *F. tricinctum* in Syrian wheat. This species is not considered as one of the main causal agents of FHB, but it was reported with a high incidence in some areas and under certain climatic conditions [14]. *F. verticillioides* has rarely been found in wheat [14]; *Fusarium* isolates similar to “*F. moniliforme*” (a now abandoned taxonomic name; the taxon was split into different species) were described in Syrian wheat kernels [15].

Table 2 shows the occurrence of *Fusarium* species in wheat samples from different provinces. *F. culmorum* was not detected in samples from Aleppo and Idlib provinces, while it was isolated from all other provinces. In the time period covered by our survey, some *Fusarium* species were limited to specific regions, such as *F. graminearum*, to Damascus and Deir Ezzor and *F. pseudograminearum* to Deir Ezzor. *F. tricinctum* was dominant in all provinces. Wheat samples from Daraa and Al-Hassakeh regions were most contaminated; *F. tricinctum*, *F. equiseti* and *F. culmorum* were identified in both regions. These results are in line with the report of El-Khalifeh *et al.*[1], who found inhomogeneous distribution of *Fusarium* spp. among Syrian provinces, too.

Comparison of the data obtained from the two years showed that the incidence of *Fusarium* spp. remained similar, but the colonization of grain with other fungal genera, particularly *Alternaria*, fluctuated to a large extent between the two years (Table 1).

The widespread presence of agents of FHB in Syria is worrying, because the legal limits for mycotoxin content in food commodities are not established in this country. We therefore assessed the potential of randomly selected *Fusarium* isolates from Syrian wheat to produce mycotoxins by chemical analysis of rice cultures. As a preliminary assessment of trichothecene production, chemotypes of the strains selected for mycotoxin analysis were assessed by PCR.

In six out of 11 *F. culmorum* strains, using the primers directed to *Tri3* (Tri3F1325/Tri3R1679), an amplification product of about 350 bp was obtained, as expected for 3Ac-DON chemotypes [16]. Five strains, amplified by the Tri7F340/Tri7R965 primers (*Tri7* gene), generated a 625 bp fragment, expected for NIV producers. The 700-bp fragment specific for the 15Ac-DON chemotype was not found in any of the tested strains. The situation is similar to Italy [17] and to other European countries, in particular England, where 3Ac-DON and NIV chemotypes dominated over the 15Ac-DON chemotype [18], and Luxemburg, where 3Ac-DON and NIV chemotypes made up 53.2% and 46.8% of all *F. culmorum* isolates, respectively [19].

All six *F. graminearum* strains were tested using primers for *Tri*12 produced amplicons of 840 bp, as expected for NIV chemotype [20]. None of the strains produced amplicons of 670 or 410 bp, expected for 15Ac-DON and 3Ac-DON chemotypes, respectively.

Nine *F. equiseti* strains were tested for the presence of the trichodiene synthase gene involved in trichothecene synthesis [21]. The presence of a 658 bp amplification product revealed the potential for trichothecene production in seven of these strains.

Mycotoxin production by ten *F. culmorum* strains, six *F. graminearum*, two *F. pseudograminearum* and six *F. equiseti* strains in rice cultures was determined by HPLC-MS/MS. The results are shown in Table 3. Four out of ten *F. culmorum* strains produced nivalenol/fusarenon X, while six, classified as the 3Ac-DON chemotype by PCR, produced high DON amounts, smaller amounts of 3Ac-DON and yet smaller amounts of 15Ac-DON. This behavior is common in strains belonging to the 3Ac-DON chemotype [22], because acetyltrichothecene esterase (a product of the gene, *Tri8*) is present in all three chemotypes; merely its activities towards C3 and C15 positions differ [23]. Similar to our results, ten *F. culmorum* strains belonging to the 3Ac-DON chemotype from France produced large amounts of DON [24]. The fact that only two among these strains produced detectable amounts of 15Ac-DON can be explained by the overall 10-times lower trichothecene amounts produced (wheat grain cultures were used instead of rice cultures). In Norway, Germany, Denmark and Hungary, all of the strains that belonged to chemotype 3Ac-DON according to PCR produced DON, too [18,25]. Co-production of DON and NIV was reported less frequently; it has been described for a few European isolates of *F. culmorum*[25] and in a recent study [26] for all *F. graminearum* isolates of the DON chemotype. We hypothesize that the co-production of DON and NIV was overlooked in studies that used culture conditions less conductive to trichothecene production and/or less sensitive analytical methods. Our results showed that chemotyping by PCR generates an oversimplified picture of trichothecene productivity in *Fusarium* spp. Because 3Ac-DON chemotype strains produced more DON than 3Ac-DON, differentiation between strong and weak DON producers would be more useful than distinction between 3Ac-DON and 15Ac-DON producers. Most NIV producers in our work also produced Fus X, which is not surprising, because Fus X is 4-acetyl-NIV. NIV producers contain active trichothecene-4-acetylase, a product of the gene, *Tri7*[27].

All six investigated *F. graminearum* strains belonged to the NIV chemotype; the PCR results were confirmed by chemical analysis of rice cultures. Similar results were reported for another area of the Middle-East/Iran [28], where the majority (46/57) of *F. graminearum* isolated from cereals possessed the NIV chemotype. The dominance of the 15Ac-DON chemotype of *F. graminearum* was reported for the Netherlands [29], England and Wales [18], Italy [30] and the USA [31], while the 3Ac-DON chemotype dominated in western Russia and Finland [32].

NIV production by *F. equiseti* was mentioned by several authors [7,33], while the production of DON by this species was reported less often [34]. Two *F. equiseti* strains in our work produced NIV (Table 3). A few *F. equiseti* strains originally appeared to produce DON, but a re-investigation did not confirm the result, indicating that *F. equiseti* does not contribute to the contamination of Syrian wheat with DON. Both *F. pseudograminearum* isolates produced DON in line with previous reports, e.g., by Monds *et al.*[35].

All investigated cultures of *F. culmorum*, *F. graminearum* and *F. pseudograminearum* and most strains of *F. equiseti* produced ZEN; the largest amounts were produced by *F. pseudograminearum* and *F. equiseti*. All *F. culmorum* strains were pathogenic on wheat (data not shown).

The study revealed that numerous toxigenic *Fusarium* species occurred in Syrian wheat. Grain from areas with higher rainfall was more often colonized with *Fusarium* and other fungal genera than grain from dry areas. Durum wheat was more susceptible to colonization than soft wheat.

## 3. Experimental Section

### 3.1. Fungal Isolation

During 2009 and 2010, 48 grain samples of 34 durum and 14 soft wheat were collected from six Syrian regions distributed in five agro-climatic zones (Figure 1) [3]. Four hundred kernels (except for a single sample from Daraa from year 2009 consisting of only 100 seeds, due to insect damage), selected randomly from each sample, were disinfected in a sodium hypochlorite solution with 2% available chlorine for 2 min, rinsed with sterile water, dried on sterile filter paper, placed in petri dishes containing potato dextrose agar (PDA, Difco, Detroit, MI, USA) supplemented with neomycin and streptomycin sulfate (100 mg/L and 200 mg/L, respectively) and incubated at 22 °C in darkness for 7 days [36].

Micromorphology of fungal isolates was examined by light microscopy [37]. All *Fusarium* isolates were sub-cultured on water agar (2% of Bacto agar, Difco) using the single spore technique. Pure cultures of *Fusarium* spp. were grown at 22 °C (12 h photoperiod) for 10 days on carnation leaf piece agar (CLA) to produce macroconidia of uniform size and form and on PDA for the morphology of the colony [38].

The frequency of each fungal genus was calculated as the percentage of the total number of kernels.

### 3.2. Genomic DNA Isolation and Qualitative PCR

DNA was extracted from *Fusarium* mycelium, harvested from 7-day-old single-spore cultures grown on PDA, using the CTAB (hexadecyl-trimethyl-ammonium bromide) method [39,40]. *F. graminearum*, *F. culmorum*, *F. equiseti*, *F. pseudograminearum*, *F. proliferatum* and *F. verticillioides* strains were identified using species-specific primers, Fg16F/Fg16R and Fc01F/Fc01R [41], FEF1/FER1 [42], Fp1-1/Fp1-2 [43], PRO1/PRO2 and VER1/VER2 [44], respectively. Amplification was done in a T3 thermocycler (Biometra, Göttingen, Germany), according to published protocols [41–44].

*F. graminearum* and *F. culmorum* strains were characterized by multiplex PCR assays to distinguish their chemotypes regarding trichothecene synthesis. For *F. culmorum*, primers amplifying parts of the *Tri3* and *Tri7* genes were used to identify 3Ac-DON, 15Ac-DON and NIV chemotypes [16]. The primer sets, *Tri3*F971/*Tri3*R1679 and *Tri3*F1325/*Tri3*R1679, identified 15Ac-DON and 3Ac-DON chemotypes, respectively, while the primer set, *Tri7*F340/*Tri7*R965, identified the NIV chemotype.

*F. graminearum* chemotypes were identified using a multiplex version of another chemotype-specific test [30]. The primers, designed in the region of the *Tri*12 gene encoding an efflux pump for trichothecenes, distinguish among three chemotypes for B trichothecene. One primer for each pair is common to all chemotypes (12CON), while the other is specific for the 15Ac-DON chemotype (12-15F), 3Ac-DON chemotype (12-3F) and the NIV chemotype (12NF) [20]. Primers Tox5/1 and Tox5/2, derived from the DNA-sequence of the trichodiene synthase gene (*Tri5*), were used to test the ability of *F. equiseti* strains to produce trichothecenes [21].

### 3.3. Mycotoxin Detection

Rice media were prepared by autoclaving 50 g polished rice with 70 mL water. Rice cultures were inoculated with 3 agar plugs (1 × 1 cm) overgrown with *Fusarium* strains and incubated at 24 °C for 4 weeks. The samples were frozen at −20°C, freeze dried for 4 days and milled.

Rice cultures were extracted and defatted, as previously described [45]; the residue corresponding to 4 g rice was dissolved in 1 mL methanol/water (1:1, *v*/*v*). The analytes were separated on a polar-modified reverse-phase HPLC column (Polaris C18-Ether, 100 × 2 mm, 3 μm particle size; Agilent, Darmstadt, Germany) kept at 40 °C with a methanol-water gradient elution (10% to 98% in 7 min, followed by washing and equilibration steps) at a flow rate of 0.2 mL/min. Mass spectrometry detection was carried out as described [45] using mass transitions for 3Ac-DON, 15Ac-DON, Fux X and ZEN, described by Klötzel *et al.*[46], and for DON and NIV, described by Rasmussen *et al.*[47]. The limits of detection were 100 μg/kg for DON, 300 μg/kg for NIV, 20 μg/kg for ZEN, 250 μg/kg for 3Ac-DON und 15Ac-DON and 250 μg/kg Fus X.

## 4. Conclusions

The mycological survey of wheat kernels from Syria revealed prevalent colonization of the grain with *Alternaria* spp. and the presence of storage fungi (e.g., *Penicillium* spp., *Aspergillus* spp.) and plant pathogenic *Fusarium* spp. in most samples. All *Fusarium* species isolated from Syrian wheat kernels are potential causal agents of FHB. PCR chemotyping and HPLC-MS analysis of rice cultures of selected *Fusarium* isolates demonstrated their potential to produce trichothecenes B and zearalenone and raised concerns about the risk of mycotoxin accumulation in Syrian wheat grain.

The incidence of *Fusarium* species in wheat grain appears low, but it has to be monitored, given the changes in agricultural policies in Syria. For example, higher irrigation rates used after the drought waves, which recently hit Syria, may increase the risk of FHB by causing a high humidity during anthesis. The increasing role of maize in crop rotation is another factor likely to increase the incidence of FHB in Syria [5].

The Syrian agricultural policy should provide guidance to agronomic practices to avoid the spread of FHB. The presence of mycotoxigenic *Fusarium* species in all wheat-growing areas in Syria raises concern about mycotoxin exposure via food grains and indicates that national legislature for the control of grain quality is indispensable.

## Figures and Tables

**Table 1:** Frequencies of fungal genera in wheat kernels collected in Syrian Provinces in 2009–2010.

Region (year)	Irrigated/rainfed	Number of samples	Number of kernels assayed	*Alternaria* (%)	*Cladosporium* (%)	*Penicillium (*%)	*Aspergillus* (%)	*Epicoccum* (%)	*Rhizopus* (%)	*Fusarium* (%)	Other genera (%)	Uninfected kernels (%)
Al-Hassakeh (2009)	Rainfed	7	2800	36.56	8.02	6.89	5.11	0.46	3.28	1.91	2.78	35.00
Daraa 1 (2009)	Rainfed	4	1600	25.35	0.38	3.94	3.90	0.06	0.63	3.08	1.56	61.10
Daraa 2 (2009)	Irrigated	1	400	3.00	0	1.00	0	0	0	0.30	1.00	94.00
Daraa 1 (2010)	Rainfed	1	400	0.75	0.25	8.00	0.75	0	0	2.00	0	89.00
Dam. rural 1 (2009)	Rainfed	1	400	12.00	0	2.00	3.00	0	8.00	0	0	75.00
Dam. rural 2 (2009)	Irrigated	4	1600	12.31	1.67	1.67	2.38	0.75	1.13	1.40	1.08	77.63
Dam. rural 1 (2010)	Rainfed	6	2400	15.04	3.71	4.58	2.71	0	3.13	0.04	2.17	68.63
Dam. rural 2 (2010)	Irrigated	8	3200	20.25	2.91	10.63	3.47	1.00	2.88	3.41	1.44	54.03
Aleppo 1 (2009)	Rainfed	3	1200	26.11	3.71	2.84	0	0.82	0	1.24	0.76	64.51
Aleppo 2 (2009)	Irrigated	1	400	35.00	11.00	3.00	1.00	1.00	3.00	1.00	2.00	42.00
Idlib (2009)	Rainfed	1	400	6.00	3.00	2.00	0	1.00	3.00	1.00	0	84.00
Deir Ezzor (2010)	Irrigated	11	4400	35.36	23.14	1.60	0.57	0.39	1.10	0.39	1.93	31.52

Dam. rural, Damascus rural.

**Table 2:** Occurrence of *Fusarium* species in wheat grain from different provinces in Syria.

Provinces	Total number of samples	Number of *Fusarium* infected samples	*Fusarium* species found (N. of isolates)
Daraa	6	5	*F. tricinctum* (15), *F. culmorum* (13), *F. equiseti* (10)
Al-Hassakeh	7	6	*F. tricinctum* (26), *F. culmorum* (7), *F. verticillioides* (6), *F. oxysporum* (5), *F. equiseti* (4), *F. semitectum* (3), *F. proliferatum* (2)
Aleppo	4	4	*F. verticillioides* (6), *F. proliferatum* (6), *F. tricinctum* (2)
Idlib	1	1	*F. tricinctum* (1), *F. verticillioides* (1)
Deir Ezzor	11	7	*F. graminearum* (5) *F. pseudograminearum* (3), *F. proliferatum* (3), *F. equiseti* (2), *F. culmorum* (1), *F. tricinctum* (1), *F. verticillioides* (1)
Damascus rural	19	7	*F. graminearum* (16), *F. culmorum* (8), *F. equiseti* (7), *F. tricinctum* (4), *F. verticillioides* (3), *F. proliferatum* (2)
	100%	62.5%	

**Table 3:** Production of mycotoxins by *Fusarium* isolates in rice cultures.

Fusarium species	Sample strain	Chemotype	Mycotoxins					

			DON (μg/g)	3Ac-DON (μg/g)	15Ac-DON (μg/g)	NIV (μg/g)	Fus X (μg/g)	ZEN (μg/g)
*F. culmorum*	F960	3Ac-DON	>100	42.2	5.7	-	-	16.6
	F961	3Ac-DON	>100	9.0	1.7	-	-	7.7
	F962	3Ac-DON	>100	9.6	1.6	-	-	17.9
	F963	NIV	5.5	-	-	>100	30.3	5.0
	F965	NIV	9.4	0.5	-	>100	52.7	0.1
	F966	3Ac-DON	>100	48.1	6.3	-	-	1.8
	F967	NIV	9.5	0.4	-	>100	>100	12.7
	F968	3Ac-DON	>100	53.5	7.2	-	-	50.4
	F969	3Ac-DON	>100	37.3	4.4	-	-	33.0
	F970	NIV	7.6	0.2	-	>100	51.8	0.6

*F. graminearum*	F1012	NIV	-	-	-	1.5	1.9	6.7
	F1014	NIV	-	-	-	1.5	1.5	6.2
	F1016	NIV	-	-	-	1.6	1.8	4.0
	F1017	NIV	-	-	-	2.9	3.0	6.0
	F1018	NIV	-	-	-	2.1	1.7	4.5
	F1022	NIV	-	-	-	2.9	2.4	3.3

*F. pseudo-graminearum*	F1029		2.6	10.3	1.0	-	-	>100
	F1030		>100	64.8	8.0	1.3	-	0.8

*F. equiseti*	F983	-	-	-	-	-	-	<0.1
	F984	Tri5 gene	-	-	-	18.6	-	13.0
	F985	Tri5 gene	-	-	-	-	-	>100
	F990	Tri5 gene	-	-	-	-	-	-
	F991	Tri5 gene	-	-	-	0.6	0.6	<0.1
	F992	Tri5 gene	-	-	-	-	-	<0.1

DON, deoxynivalenol; NIV, nivalenol; 3Ac-DON, 3-acetyl-deoxynivalenol; Fus X, fusarenon X; ZEN, zearalenone.
